# Clinical benefit of systemic therapies for recurrent ovarian cancer—ESMO-MCBS scores

**DOI:** 10.1016/j.esmoop.2021.100229

**Published:** 2021-08-07

**Authors:** K.E. Broekman, M. van Kruchten, H. van Tinteren, C. Sessa, M. Jalving, A.K.L. Reyners

**Affiliations:** 1 Department of Medical Oncology, University of Groningen, University Medical Center Groningen, Groningen, The Netherlands; 2 Trial and Data Center, Princess Maxima Center for Pediatric Oncology, Utrecht, The Netherlands; 3 Oncology Institute of Southern Switzerland, Ospedale San Giovanni, Bellinzona, Switzerland

**Keywords:** clinical benefit, ovarian cancer, ESMO-MCBS, chemotherapy, targeted therapy

## Abstract

**Background:**

Licensed systemic treatment options for platinum-sensitive recurrent ovarian cancer are platinum-based chemotherapy and maintenance treatment with bevacizumab and poly (ADP-ribose) polymerase inhibitors. For platinum-resistant disease, several non-platinum options are available. We aimed to assess the clinical benefit of these treatments according to the European Society of Medical Oncology (ESMO)-Magnitude of Clinical Benefit Scale (MCBS).

**Materials and methods:**

A PubMed search was carried out including all studies evaluating systemic treatment of recurrent epithelial ovarian cancer, from 1990 onwards. Randomised trials with an adequate comparator and design showing a statistically significant benefit of the study arm were independently scored by two blinded observers using the ESMO-MCBS.

**Results:**

A total of 1127 papers were identified, out of which 61 reported results of randomised trials of sufficient quality. Nineteen trials showed statistically significant results and the studied treatments were graded according to ESMO-MCBS. Only three treatments showed substantial benefit (score of 4 on a scale of 1-5) according to the ESMO-MCBS: platinum-based chemotherapy with paclitaxel in the platinum-sensitive setting and the addition of bevacizumab to chemotherapy in the platinum-resistant setting. The WEE1 inhibitor adavosertib (not licensed) also scores a 4, based on a recent small phase II study. Assessment of quality-of-life data and toxicity using the ESMO-MCBS showed to be complex, which should be taken into account in using this score for clinical decision making.

**Conclusion:**

Only a few licensed systemic therapies for recurrent ovarian cancer show substantial clinical benefit based on ESMO-MCBS scores. Trials demonstrating overall survival benefit are sparse.

## Highlights

• Benefit of systemic treatments for recurrent ovarian cancer was scored on the ESMO-MCBS.
• From 1127 identified papers, only 3 trials out of 19 that were suitable for grading showed substantial clinical benefit.
• Trials demonstrating overall survival benefit are sparse.

## Introduction

First-line therapy for advanced epithelial ovarian cancer (EOC) consists of debulking surgery and platinum-based chemotherapy. EOC is usually diagnosed at an advanced stage, which has a poor prognosis with the majority of women eventually developing recurrent disease. Ovarian cancer-specific survival at 5 years is 40% for stage III disease and only 20% for stage IV disease.^1^ Treatment options and prognosis differ depending on the interval between first-line treatment and relapse (the platinum-free interval, PFI), with the highest chance of response to reintroduction of platinum-based chemotherapy in patients with a PFI of >12 months. Patients with relapse occurring within 6 months of platinum-based chemotherapy were historically considered platinum-resistant. Recently, the strict definition of platinum-resistant disease was abandoned. It is recognised that the time elapsed since the last platinum chemotherapy represents a continuum of probability of response to further chemotherapy. Treatment options mentioned in guidelines for this setting have limited response rates (RRs) in patients with a short PFI and most patients die of their disease within 1 year.^2^ Therapies licensed for treatment of recurrent ovarian cancer, besides carboplatin and paclitaxel, are gemcitabine and bevacizumab (in combination with platinum), liposomal doxorubicin with or without trabectedin, treosulfan, melphalan, topotecan, etoposide and the poly (ADP-ribose) polymerase (PARP) inhibitors olaparib, niraparib and rucaparib. Guidelines mention these drugs as treatment options and advise that the treatment regimen should be chosen based on platinum sensitivity, previously received treatments, *BRCA* mutation status and physician and patients' preferences.^2^^,^^3^ The European Society of Medical Oncology (ESMO)-Magnitude of Clinical Benefit Scale (MCBS) is an instrument to score the clinical benefit of anticancer therapies taking into account efficacy, quality of life (QoL) and toxicity.^4^ We aimed to assess the clinical benefit of systemic therapies in recurrent ovarian cancer according to the ESMO-MCBS.

## Materials and methods

### Search strategy and selection

A PubMed search was carried out in 2019 and updated in March 2021 using the following Medical Subject Headings (MeSH) terms: ‘recurrent’ OR ‘refractory’ AND ‘ovarian’ OR ‘ovary’ AND ‘neoplasm’ OR ‘cancer’ OR ‘carcinoma’; the search was limited to trials in humans. Studies evaluating systemic treatment in recurrent EOC patients were screened, from the introduction of taxanes in 1990 onwards.

Titles and abstracts were screened independently by two observers (KEB and MvK) and categorised into three categories: category I contained large phase III randomised controlled trials (RCTs) in the target population with an adequate comparator and a primary endpoint of overall survival (OS) or progression-free survival (PFS); category II for other studies that could potentially be scored on the ESMO-MCBS; and category III for studies that did not meet the inclusion criteria for scoring (e.g. wrong population, surgical interventions only, review articles, retrospective studies and case series). After all articles had been assigned to a category, the assessments of the two independent observers were compared and conflicting results were resolved at a consensus meeting. Studies in category I and II were analysed in more detail by reading the full paper, assessing study methodology, patient numbers, study design, endpoints and outcome. From category II, only randomised studies comparing a new agent or combination to a clinically relevant comparator were included, and thus single-arm studies and studies evaluating different dose regimens of the same drug were excluded from the analysis. Non-platinum-based chemotherapy was inferior to platinum-based chemotherapy in the MITO-8 study,^5^ and platinum-based chemotherapy was therefore considered the preferred comparator treatment for the included trials in the platinum-sensitive setting. For the platinum-resistant setting, any treatment option mentioned in the ESMO or American Society of Clinical Oncology guidelines was considered an adequate comparator.

Finally, references of included studies and relevant guidelines were checked to identify relevant publications not retrieved by the search strategy. All included studies showing a statistically significant benefit of the study arm were graded according to the ESMO-MCBS.

### Grading according to ESMO-Magnitude of Clinical Benefit Scale

The ESMO-MCBS can be used to rank the value of systemic therapies based on reported relative and absolute benefits in terms of improved survival (PFS, disease-free survival, OS) and better survival (e.g. QoL, toxicity). For palliative treatments, as is the case in recurrent ovarian cancer, there are several MCBS evaluation forms available and the correct form is chosen based on the primary endpoint used in the study (OS, PFS, QoL, toxicity or RR) and the duration of survival in the control group.^4^ Palliative treatments are graded 1-5, where grades 4 and 5 represent a substantial clinical benefit. The preliminary score for palliative treatments is upgraded when the study treatment shows an improvement in QoL or a reduction in grade 3-4 toxicities impacting daily well-being. For treatments that only show a benefit in PFS, but not OS, the preliminary score is downgraded if the study treatment has increased toxicity or does not demonstrate improvement in QoL.^4^^,^^6^ The MCBS allows for scoring of clinical benefit in a maximum of three prespecified subgroups, provided adjustments for multiple comparisons are taken into account.

## Results

### Search results and trial selection

**Figure 1:**
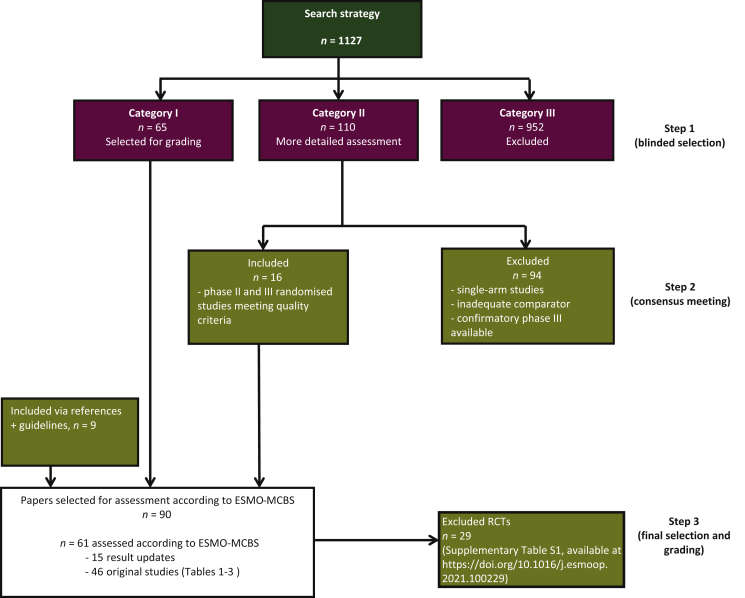
CONSORT diagram. Flow chart showing the search strategy and steps in selection and grading of trials. ESMO, European Society of Medical Oncology; MCBS, Magnitude of Clinical Benefit Scale; RCT, randomised controlled trial

Our search retrieved 1127 studies in ovarian cancer published since 1990. There was an excellent overall agreement between the two observers (98%) when categorising the studies into the three specified groups. A flow chart indicating the selection procedure is shown in Figure 1. Sixty-five studies were directly included based on the abstract (listed as category I by both observers). Nine hundred and fifty-two studies were directly excluded based on the abstract (category III). The remaining 110 studies (category II) were reviewed in more detail and discussed at a consensus meeting. Sixteen studies from category II matched criteria for inclusion in the analysis. After detailed assessment of full-text articles, 29 RCTs from category I and II were excluded because of an inadequate comparator, premature termination of the trial or the availability of a higher-quality trial studying the same treatment (Supplementary Table S1, available at https://doi.org/10.1016/j.esmoop.2021.100229). Finally, nine additional studies were included after checking relevant guidelines and references.

In total, 61 publications reporting the results of 46 original clinical trials were assessed. Fourteen phase III studies and five randomised phase II studies with statistically significant results were graded for clinical benefit using the ESMO-MCBS (Tables 1 and 2). The remaining 27 studies did not report a statistically significant result and were therefore not graded on the ESMO-MCBS (Table 3).

**Table 1:** Phase III studies scored for clinical benefit (*n* = 14)

Treatment	Study name	Subgroups	*n*	Control	Primary endpoint	PFS control	PFS gain	HR	OS control	OS gain	OS HR	ORR	Toxicity/QoL adjustment	MCBS score (form)	Ref.
Platinum-sensitive recurrence															
Carboplatin/PLD	CALYPSO		976	Carboplatin plus paclitaxel	PFS (non-inferiority)	9.4	1.9	0.82 (0.72-0.94)	30.7	2.3	0.99 (0.85-1.16)		Global QoL equal but better scores on subscales for carboplatin/PLD (less neurotoxicity, better body image; early discontinuation in 6% versus 15%)	3 (2c)	^8^^,^^9^
Paclitaxel plus platinum-based chemotherapy	ICON4/AGO-OVAR 2.2		802	Platinum monotherapy (carboplatin or cisplatin)	OS				24	5	0.82 (0.69-0.97)			4 (2a)	^7^
Carboplatin plus gemcitabine			356	Carboplatin	PFS	5.8	2.8	0.72 (0.58-0.90)	17.3	0.7	0.96 (0.75-1.23)		−1 No QoL benefit and no OS benefit	2 (2b)	^10^
Carboplatin/PLD/bevacizumab^b^			682	Carboplatin/gemcitabine/bevacizumab	PFS	11.7	1.7	0.81 (0.68-0.91)	27.8	4.1	0.81 (0.67-0.98)			1 (2b)	^11^
Carboplatin doublet plus bevacizumab	MITO16b/MANGO-OV2/ENGOT-ov17		406	Carboplatin doublet	PFS	8.8	3	0.51 (0.41-0.65)						3 (2b)	^12^
Maintenance therapy after response to second-line platinum-based chemotherapy															
Cediranib	ICON6	- ITT unselected	282	Placebo	PFS	8.7	2.3	0.56 (0.44-0.72)			0.86 (0.67-1.11)		−1 No QoL benefit and no OS benefit	1 (2b)	^21^^,^^28^
Niraparib		- ITT - *gBRCA* mutation - Non-*gBRCA* mutation (including HRD+) - HRD+	553 203 350 162	Placebo	PFS	— 5.5 3.9 3.8	— 14.5 5.4 9.1	— 0.27 (0.17-0.41) 0.45 (0.34-0.61) 0.38 (0.24-0.59)						3 (2b) 3 (2b)	^17^
Rucaparib (ITT)	ARIEL3	- ITT - *BRCA* mutation - HRD (including *BRCA*-mutated group)	564 196 354	Placebo	PFS	5.4 5.4 5.4	5.4 11.2 8.2	0.36 (0.30-0.45) 0.23 (0.16-0.34) 0.32 (0.24-0.42)						3 (2b) 3 (2b) 3 (2b)	^18^
Olaparib (tablets)	SOLO2	- ITT All had *gBRCA*1/2 mutation	295	Placebo	PFS	5.5	13.6	0.30 (0.22-0.41)	38.8	12.9	0.74 (0.54-1.00)		−1 for no OS benefit	2 (2b)	^20^^,^^27^
Addition of bevacizumab to carboplatin plus gemcitabine	OCEANS		484	Carboplatin plus gemcitabine	PFS	8.4	4	0.48 (0.39-0.61)	32.9	0.7	0.95 (0.77-1.18)		−1 No QoL benefit and no OS benefit	2 (2b)	^22^^,^^23^
Platinum-resistant recurrence															
Trabectedin plus PLD^a^			672	PLD	PFS	5.8	1.5	0.79 (0.65-0.96)	18.9	3.3	0.86 (0.72-1.02)		−1 No QoL benefit and no OS benefit	2 (2b)	^34^^,^^35^
Addition of bevacizumab to standard chemotherapy (either PLD, paclitaxel or topotecan)	AURELIA		361	Standard chemotherapy (either PLD, paclitaxel or topotecan)	PFS	3.4	3.3	0.48 (0.38-0.60)	13.3	3.3	0.85 (0.66-1.08)		+1 QoL benefit	4 (2b)	^30^^,^^31^
Trebananib plus paclitaxel^a^	TRINOVA-1		919	Paclitaxel	PFS	5.4	1.8	0.66 (0.57-0.77)	18.3	1	0.95 (0.85-1.11)		−1 No QoL benefit and no OS benefit	2 (2b)	^36^
Topotecan^a^			235	Paclitaxel	ORR							ORR 21%		2 (3)	^45^

*gBRCA*, germline *BRCA*; HR, hazard ratio; HRD, homologous recombination deficiency; ITT, intention to treat; MCBS, Magnitude of Clinical Benefit Scale; ORR, objective response rate; OS, overall survival; PFS, progression-free survival; PLD, pegylated liposomal doxorubicin; QoL, quality of life.

a Trial did also include platinum-sensitive patients.

b Trial not scored for OS (form 2a) as OS was exploratory and not corrected for multiple testing.

**Table 2:** Phase II studies scored for clinical benefit (*n* = 5)

Treatment	Study name	Subgroups	*n*	Control	Primary endpoint	PFS control	PFS gain	HR	OS control	OS gain	OS HR	Toxicity/QoL adjustment	MCBS score	Ref.
Maintenance therapy after response to second line platinum-based chemotherapy														
Olaparib (capsules)	STUDY19	- ITT - *BRCA*-mut - *BRCA*-w/t	265 136 118	Placebo	PFS	4.8 4.3 5.5	3.6 6.9 1.9	0.35 (0.25-0.49) 0.18 (0.10-0.31) 0.54 (0.34-0.85)	27.8 30.2 26.6	2 4.7 −2.1	0.73^a^ (0.55-0.95) 0.62^a^ (0.42-0.93) 0.84 (0.57-1.25)	−1 No QoL benefit and no OS benefit	2 (2b) 2 (2b) 2 (2b)	^19^^,^^24^
Olaparib (capsules) during carboplatin/paclitaxel followed by olaparib maintenance		- ITT unselected	173	Carboplatin/paclitaxel followed by no further treatment	PFS	9.6	2.6	0.51 (0.34-0.77)					2 (2b)	^25^
Platinum-resistant recurrence														
Sorafenib plus topotecan	TRIAS		185	Toptecan plus placebo	PFS	4.4	2.3	0.60 (0.43-0.83)					3 (2b)	^33^
Berzosertib plus gemcitabine			70	Gemcitabine	PFS	3.4	1.9	0.57 (0.33-0.98)	9.9	3.8	0.84 (0.53-1.32)	−1 No QoL benefit and no OS benefit	2 (2b)	^37^
Adavosertib plus gemcitabine			99	Gemcitabine	PFS	3	1.6	0.55 (0.35-0.90)	7.2	4.2	0.56 (0.35-0.91)		4 (2a)	^32^

HR, hazard ratio; ITT, intention to treat; MCBS, Magnitude of Clinical Benefit Scale; OS, overall survival; PFS, progression-free survival; QoL, quality of life; w/t, wild type.

a *P* value did not meet prespecified study criteria for significant OS result.

**Table 3:** Studies without statistically significant benefit (*n* = 27)

Treatment	Study name	*n*	Control	Design	Primary endpoint	PFS control	PFS gain	HR	OS control	OS gain	OS HR	ORR	Toxicity/QoL	ESMO grading	Ref.
Platinum-sensitive recurrence															
Farletuzumab with carboplatin/taxane		1100	Placebo with carboplatin/taxane	Phase III RCT	PFS	9.0	0.7	0.99 (0.81-1.21)						Not significant	^13^
Carboplatin/topotecan		550	Carboplatin plus physician choice either paclitaxel, gemcitabine or PLD	Phase III	PFS	10	0	Not provided						Not significant	^14^
Non-platinum-based CT	MITO-8	215	Platinum-based CT	Phase III RCT	OS	9	−4	Not provided	24.5	−2.7	Not provided			PFS control arm significantly better	^5^
Carboplatin/paclitaxel micellar		789	Carboplatin/paclitaxel (conventional)	Phase III non-inferiority RCT	Non-inferiority for PFS	10.1	0.2	0.86 (0.72-1.03)					Increased neutropenia, no difference in neurotoxicity	No score (no improvement in QoL or symptoms)	^15^
Maintenance therapy after response to second-line platinum-based chemotherapy															
Vismodegib maintenance after complete response to second- or third-line chemotherapy		104	Placebo	Phase II RCT	PFS	5.8	1.7	0.79 (0.46-1.35)						Not significant	^29^
Carboplatin/paclitaxel plus bevacizumab	GOG-0213	674	Carboplatin/paclitaxel plus placebo	Phase III RCT	OS				37.3	4.9	0.83 (0.68-1.01)			Not significant	^26^
Platinum-resistant recurrence															
PLD^a^		474	Topotecan	RCT	PFS	3.9	−0.2	NS	13.8	0.69	0.82 (0.68-1.00)			Not significant	^46^^,^^47^
Epidoxorubicin plus paclitaxel^a^		234	Paclitaxel	Phase III RCT	OS				14.0	−2.0	NS			Not significant	^48^
PLD		195	Gemcitabine	Phase III RCT	PFS	3.1	0.5	NS						Not significant	^49^
PLD^a^		153	Gemcitabine	Phase III RCT	TTP	3.7	0.9	NS						Not significant	^50^
Topotecan/etoposide (TE) or topotecan/gemcitabine		502	Topotecan	Phase III RCT	OS				17.2	0.6 (TE)	1.18 (0.90-1.53)			Not significant	^51^
Canfosfamide^a^		461	PLD or topotecan	Phase III RCT	OS				13.5	−5.0	1.71			Control arm significantly better	^52^
Pertuzumab plus gemcitabine		130	Gemcitabine plus placebo	Phase II RCT	PFS	2.9	0	0.66 (0.43-1.03)						Not significant	^53^
Pertuzumab plus carboplatin and either paclitaxel or gemcitabine		149	Carboplatin and either paclitaxel or gemcitabine	Phase II RCT	PFS	8.6	−0.7	1.16 (0.90-1.49)						Not significant	^54^
Paclitaxel/carboplatin OR paclitaxel/topotecan		165	Paclitaxel	Phase II RCT	PFS	3.7	1.1 1.7	0.92, NS 0.95, NS						Not significant	^55^
Olaparib 200 mg or 400 mg^a^		97	PLD	Phase II RCT	PFS	7.1	NA	0.88 (0.62-1.28)						Not significant	^56^
Patupilone		829	PLD	Phase III RCT	OS				12.7	0.5	0.93 (0.79-1.09)			Not significant	^57^
Docetaxel plus vandetanib		129	Docetaxel	RCT	PFS	3.5	−0.5	1.01 (0.79-1.27)						Not significant	^58^
Seribantumab plus paclitaxel		223	Paclitaxel	RCT	PFS	3.7	0.1	1.03 (0.74-1.43)						Not significant	^59^
Motolimod plus PLD		297	PLD	Phase II RCT	PFS	5.2	−0.4	1.21	18.9	−0.8	1.22			Not significant	^60^
Oncolytic reovirus plus paclitaxel		108	Paclitaxel	Phase II RCT	PFS	4.3	0.1	1.11 (0.78-1.59)						Not significant	^61^
Pazopanib plus paclitaxel		106	Paclitaxel	Phase II RCT	PFS	6.2	1.3	0.84 (0.57-1.22)	23.3	−2.6	1.04 (0.60-1.79)			Not significant	^62^
Cabozantinib plus paclitaxel		174	Paclitaxel	RCT	PFS	5.5	−0.2	1.11 (0.77-1.61)	Not reached		2.27 (1.17-2.63)			Control arm significantly better OS	^63^
Olaratumab plus PLD		123	PLD	Phase II RCT	PFS	4.0	0.2	1.04 (0.70-1.56)	16.2	0.4	1.1 (0.70-1.71)			Not significant	^64^
Linsitinib (two different regimens) plus paclitaxel		152	Paclitaxel	Phase I/II RCT	PFS	5.6	−1.4	1.2 (0.75-1.91)						Not significant	^65^
Alisertib plus paclitaxel		191	Paclitaxel	Phase I/II RCT	PFS	4.7	2.0	0.75 (0.58-0.96)						Not significant	^66^
Carboplatin plus guadecitabine		100	Physician choice PLD, topotecan, paclitaxel or gemcitabine	Phase II RCT	PFS	2.1	1.6	0.69 (0.46-1.0)						Not significant	^67^

HR, hazard ratio; ITT, intention to treat; MCBS, Magnitude of Clinical Benefit Scale; NS, not significant; OS, overall survival; PFS, progression-free survival; PLD, pegylated liposomal doxorubicin; QoL, quality of life; RCT, randomised controlled trials; TTP, time to progression.

a Trial did also include platinum-sensitive patients.

### Clinical benefit of treatment in platinum-sensitive recurrent ovarian cancer

#### Platinum-based chemotherapy

Nine randomised phase III studies evaluating chemotherapy in platinum-sensitive disease in a total of 5470 patients were assessed for clinical benefit. Of these nine trials, five (55%) showed significant improvement of either PFS (3/9), OS (1/9) or QoL (1/9) (Table 1).

With regard to the platinum-based options, the highest clinical benefit (ESMO-MCBS grade 4) was obtained for the combination of paclitaxel plus platinum-based chemotherapy.^7^ In the ICON4 trial, comparing the combination of platinum-based chemotherapy with paclitaxel to carboplatin or cisplatin monotherapy, the combination resulted in a 5-month improvement of OS {median OS 29 versus 24 months; hazard ratio (HR) 0.82 [95% confidence interval (CI) 0.69-0.97]}. Carboplatin/paclitaxel was used as the comparator treatment in trials carried out after ICON4. Treatment with carboplatin plus pegylated liposomal doxorubicin (PLD) has a lower ESMO-MCBS grade (grade 3) than carboplatin/paclitaxel, but the comparator arm in these two studies was different. Namely, while carboplatin/paclitaxel was compared to platinum monotherapy in the ICON4 trial, carboplatin plus PLD was compared to carboplatin/paclitaxel in the CALYPSO study.^8^ In this non-inferiority study, PFS was longer for carboplatin/PLD as compared to carboplatin/paclitaxel [median PFS 11.3 versus 9.4 months; HR 0.82 (0.72-0.94)]. Global QoL scores were equal, but there was an improvement in various symptom subscales with carboplatin/PLD, including peripheral neuropathy, global chemotherapy side-effects and impact on body image^9^ resulting in a grade 3 score (form 2c). Early treatment discontinuation was also less frequent for carboplatin/PLD (6%) than for carboplatin/paclitaxel (15%).

**Figure 2:**
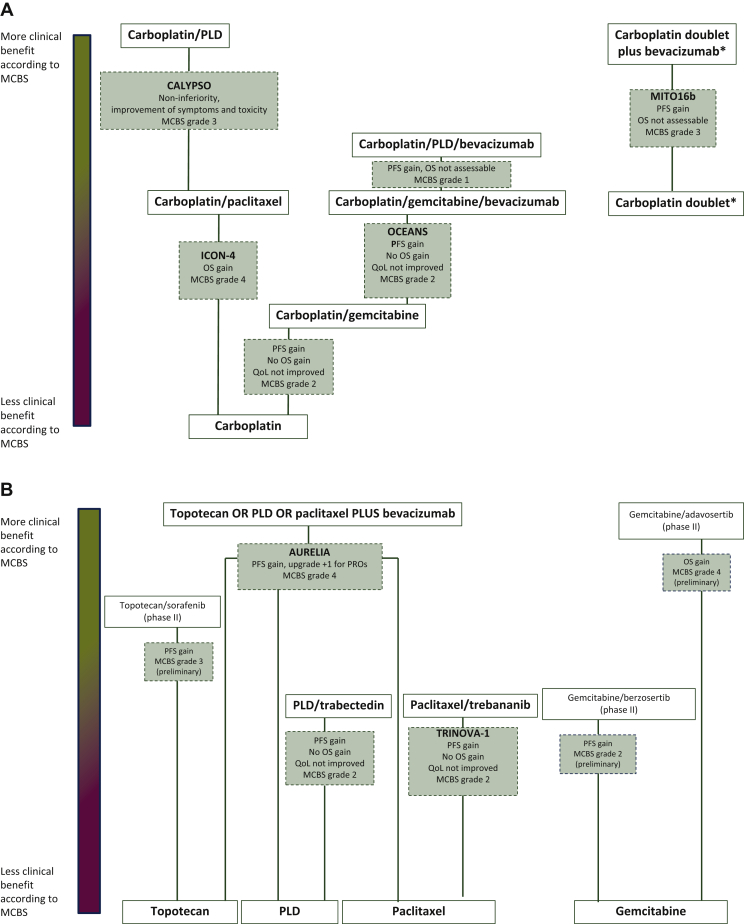
Relative difference in MCBS between comparators in platinum-sensitive (A) and platinum-resistant (B) ovarian cancer. An overview of the relative benefit between the different arms and comparators studied and graded for the platinum-sensitive and -resistant setting is provided. The distance between two comparators is representative for the MCBS grading with larger distance representing more benefit according to MCBS for the intervention versus. comparator. ∗In the MITO16b study, carboplatin doublets studied were carboplatin with either paclitaxel, gemcitabine or liposomal doxorubicin. MCBS, Magnitude of Clinical Benefit Scale; OS, overall survival; PFS, progression-free survival; PLD, liposomal doxorubicin; PRO, patient-reported outcomes; QoL, quality of life.

The remaining three trials with significant results investigated carboplatin doublet chemotherapy plus bevacizumab (versus carboplatin doublet chemotherapy), carboplatin plus gemcitabine (versus carboplatin monotherapy) and carboplatin plus PLD plus bevacizumab (versus carboplatin/gemcitabine/bevacizumab), which all showed an improved PFS over the comparator, but did not result in OS benefit and showed no improved QoL.^10^, ^11^, ^12^ This qualifies as only limited clinical benefit with ESMO-MCBS grades of 3, 2 and 1, respectively. An overview of the relative benefit between the different arms and comparators studied and graded for the platinum-sensitive setting is provided in Figure 2A.

Studies with negative results included the addition of the folate receptor antibody farletuzumab to carboplatin plus taxane,^13^ carboplatin plus topotecan^14^ and carboplatin plus micellar paclitaxel.^15^

In conclusion, for platinum-sensitive recurrence, the first-choice treatment is a carboplatin-based combination regimen. Direct comparison in the CALYPSO trial demonstrated that carboplatin plus PLD has a slightly better toxicity profile than carboplatin/paclitaxel. Carboplatin/paclitaxel renders comparable OS^16^ but may result in more toxicity and early discontinuation.

#### Maintenance therapy after platinum-based chemotherapy

Nine randomised studies in 3394 patients evaluated maintenance therapy after response to platinum-based chemotherapy for recurrent disease. Seven of nine trials (78%) reported significant results: three phase III RCTs evaluating maintenance therapy with PARP inhibitors,^17^, ^18^, ^19^, ^20^ one phase III RCT assessing maintenance with cediranib, a tyrosine kinase inhibitor targeting the vascular endothelial growth factor receptor,^21^ one phase III RCT studying bevacizumab maintenance^22^^,^^23^ and two phase II RCTs with the PARP inhibitor olaparib (capsules).^24^^,^^25^ In six studies, maintenance therapy was initiated only after response to platinum-based chemotherapy for recurrent disease. Three studies were conceptually different because the investigational agent was started concomitantly with chemotherapy and continued thereafter (one study with olaparib^25^ and two studies with bevacizumab^22^^,^^23^^,^^26^).

The phase II STUDY19 compared olaparib capsules with placebo: there was no significant improvement in OS or QoL for the intention-to-treat (ITT) population or for the prespecified *BRCA*-mutated subgroup (germline or somatic). This resulted in the preliminary score of 3 for the observed PFS benefit which was downgraded to a grade 2 in both populations.^24^ Treatment with olaparib tablets was assessed in the phase III SOLO2 trial, in which all included patients had a germline *BRCA* (*gBRCA*) mutation.^20^ The mature OS results were recently published.^27^ The observed OS benefit in the ITT population (*n* = 295) did not reach statistical significance with an HR of 0.74 (0.54-1.00; *P* = 0.054), which results in a downgrade to grade 2 for the ITT *gBRCA*-mutated population. Remarkably, a pre-planned sensitivity analysis in patients with Myriad-confirmed *gBRCA* mutation (*n* = 286 of the total 295 patients) did show a statistically significant OS benefit of 15 months [HR 0.71 (0.52-0.97); *P* = 0.0306] compared to placebo (OS 37.4 months). However, because there was no correction for multiple testing, this analysis was considered exploratory and the results were not scored on the ESMO-MCBS.

Treatment with the PARP inhibitors niraparib and rucaparib resulted in moderate benefit according to ESMO-MCBS (grade 3) when compared to placebo, all based on PFS as primary endpoint. This score was the same for the benefit in the unselected ITT population as well as the prespecified subgroup analyses based on *BRCA* status, although the included study populations differed somewhat between studies. The absolute benefit was largest for patients with a *BRCA* mutation with a gain in PFS of 11 and 15 months for rucaparib and niraparib, respectively. For niraparib, analysis of PFS in the subgroups of patients with and without a *gBRCA* mutation was predefined. The non-*gBRCA* subgroup included patients with homologous recombination deficiency (HRD) (162/350; 46%), of which a minority had a somatic *BRCA* (*sBRCA*) mutation (47/350; 13%).^17^ Separate analyses in the patients with HRD or *sBRCA*-mutated tumours were exploratory and therefore not scored on the MCBS. For rucaparib, both the analyses in the subgroup of patients with HRD-associated disease (*n* = 354, including *n* = 196 with *BRCA* mutation) and the *BRCA*-mutated subgroup (germline and somatic) were predefined and therefore scored.^18^

Maintenance treatment with cediranib improved PFS by 2 months compared to placebo in the ICON6 trial, but not OS,^21^^,^^28^ not qualifying as substantial benefit (ESMO-MCBS grade 1). Maintenance therapy with vismodegib has also been evaluated but did not result in improvement of PFS or OS.^29^

The benefit of bevacizumab maintenance therapy after platinum-based chemotherapy combined with bevacizumab was assessed in the OCEANS trial and GOG-0213 trial. In the OCEANS trial, addition of bevacizumab to carboplatin/gemcitabine resulted in a PFS gain only, without improvement of OS or QoL, qualifying as only limited clinical benefit with an ESMO-MCBS grade 2.^22^^,^^23^ Addition of bevacizumab to carboplatin/paclitaxel did not render a statistically significant benefit and was therefore not graded.^26^

In conclusion, despite the promise and biological rationale of targeted treatment with PARP inhibitors after response to platinum-based chemotherapy, in the recurrent setting a substantial clinical benefit based on OS gain cannot be confirmed for olaparib (tablets) in patients with a *gBRCA* mutation, with a score of 2 according to the ESMO-MCBS. The benefit was moderate for other PARP inhibitors based on a PFS benefit. As of yet, OS results for other PARP inhibitors than olaparib are awaited which may result in either a downgrade or an upgrade of the preliminary score. Maintenance therapy with bevacizumab does not provide moderate or substantial benefit according to ESMO-MCBS in patients with platinum-sensitive recurrence.

### Clinical benefit of treatment in the platinum-resistant setting

A total of 8137 patients with platinum-resistant ovarian cancer were treated in 28 randomised studies included in our analysis, out of which only 6 trials (21%) resulted in significant improvement of either PFS (5/6) or OS (1/6).

Two trials showed substantial benefit (ESMO-MCBS grade 4) in this setting. In the AURELIA study, the addition of bevacizumab to standard chemotherapy with either PLD, paclitaxel or topotecan was compared to PLD, paclitaxel or topotecan monotherapy.^30^ The addition of bevacizumab to physician-choice chemotherapy resulted in a 3.3-month improvement of PFS [median PFS 6.7 versus 3.4 months; HR 0.48 (96% CI 0.38-0.60)]. Although OS was similar in both groups, the addition of bevacizumab did result in improvement in a predefined analysis of a subscale of patient-reported outcomes with a 15% improvement of abdominal/gastrointestinal (GI) symptoms.^31^ The preliminary score of 3 was upgraded to an ESMO-MCBS grade 4 based on this QoL benefit. The combination of the WEE1 inhibitor adavosertib with gemcitabine also improved both PFS and OS compared to gemcitabine monotherapy [median OS 7.2 versus 11.4 months; HR 0.56 (96% CI 0.35-0.91)].^32^ However, this was assessed in a phase II study only and a difference in prognostic baseline factors between groups might bias the results of this small trial. Therefore, the ESMO-MCBS score of 4 should be considered as preliminary.

One trial showed moderate benefit (grade 3) of sorafenib plus topotecan compared to topotecan alone, with a statistically significant 2.3-month PFS gain, evaluated in a phase II study.^33^ Other treatments, such as PLD plus trabectedin compared to PLD monotherapy,^34^^,^^35^ trebananib plus paclitaxel compared to paclitaxel monotherapy^36^ and the ataxia telangiectasia and Rad3-related protein (ATR) inhibitor berzosertib plus gemcitabine compared to gemcitabine monotherapy,^37^ had only limited benefit (grade 2). An overview of the relative benefit between the different arms and comparators studied and graded for the platinum-resistant setting is provided in Figure 2B.

Therapies studied in the platinum-resistant setting that were unable to improve PFS or OS are listed in Table 3. This includes studies that compared the different agents used in the comparator arm of the AURELIA trial (PLD, paclitaxel or topotecan), making it difficult to recommend one of these agents over the other. Therefore, due to lack of convincing evidence, the choice of therapy will largely be based on previously received treatments and physician and patients' preferences regarding scheduling and side-effects.

In conclusion, topotecan, PLD and paclitaxel are used for treatment of platinum-refractory ovarian cancer but there are no studies comparing these therapies to best supportive care. Addition of bevacizumab to these agents improved PFS and some symptoms; however, no OS benefit was observed. The WEE1 inhibitor adavosertib combined with gemcitabine has a preliminary score of 4 based on an OS benefit observed in a phase II study; this drug has not yet been licensed. Other treatments or combinations have shown only limited benefit compared to monotherapy with topotecan, PLD or paclitaxel.

## Discussion

Analysis of the clinical benefit of systemic therapies for recurrent ovarian cancer using the ESMO-MCBS shows substantial benefit of only a few licensed treatments: carboplatin with either paclitaxel or PLD in the platinum-sensitive setting and the addition of bevacizumab to topotecan, paclitaxel or PLD in the platinum-resistant setting. Considering that over 18 000 patients were enrolled in randomised clinical trials in ovarian cancer over the last three decades, this result is disappointing. This is in line with the moderate improvement of 5-year survival rate in advanced ovarian cancer of only 2% from 1983 to 2012.^38^ Most of the studied treatments were tested in large RCTs and did not improve PFS, OS or QoL. Therefore, the lack of benefit is more likely due to the inefficacy of the drugs rather than due to badly designed trials. Treosulfan, melphalan, paclitaxel and etoposide received a broad label for treatment of ovarian cancer decades ago based on registration trials that included patients with newly diagnosed EOC. These drugs were not tested specifically in the recurrent setting in large trials; therefore, these were not included in the current analysis and not scored on the ESMO-MCBS.

Based on their mechanism of action, PARP inhibitors are expected to especially benefit patients with homologous recombination-deficient tumours, such as *BRCA*-mutated ovarian cancer.^39^ HRD can also be caused by other alterations in the homologous recombination repair pathway.^40^ Based on the recently published mature OS results, maintenance treatment with olaparib (tablets) did not yield a substantial clinical benefit (grade 2) for patients with a *gBRCA* mutation. Surprisingly, a sensitivity analysis in the population with a Myriad-confirmed *gBRCA* mutation, which included only nine additional patients, did result in a statistically significant OS benefit. However, because correction for multiple testing was not applied, this sensitivity analysis is considered exploratory and its results were not scored on the ESMO-MCBS. Grading of the other licensed PARP inhibitor therapies (niraparib and rucaparib) on the ESMO-MCBS labels their benefit as only ‘moderate’ with a score of 3. This score was not higher in the prespecified subgroup of patients with a *BRCA* mutation compared to the unselected ITT population. The PFS gain in the ITT population for rucaparib, and in the non-*gBRCA*-mutated patients for niraparib was, however, numerically lower than in the other subgroups. It is, therefore, essential to understand the benefit of PARP inhibitors in patients with *BRCA* wild-type disease and absence of HRD, since the gain observed for the ITT population is likely to be largely driven by the inclusion of patients with either HRD tumours or *BRCA*-mutated tumours. This supports restricting use of olaparib tablets to this group of patients.^41^ Niraparib and rucaparib received a broad label, which includes treatment of patients irrespective of *BRCA* mutation status. The registration trial for olaparib capsules (STUDY19) also included *BRCA*-negative patients, but it received a restricted licensed indication only in patients with a *BRCA* mutation. Later, an updated OS analysis indeed only showed a numerical OS gain in patients with a *BRCA* mutation supporting this.^19^ This OS benefit did not meet the prespecified criterion for statistical significance; therefore, the preliminary ESMO-MCBS score of 2 was not upgraded. For olaparib tablets (SOLO2), the ITT population only included patients with a confirmed *BRCA* mutation. Remarkably, registration was partly based on data from the ITT analysis of STUDY19, leading to a broader licensed label for olaparib tablets, including treatment of patients without a *BRCA* mutation.^42^

Despite the use of debulking surgery for selected patients with recurrent disease, given the low chance of cure for these patients, we considered chemotherapy after debulking surgery for recurrent ovarian cancer a palliative treatment and therefore used ESMO-MCBS forms 2 or 3. For treatments that only show a PFS benefit, without an OS benefit, the ESMO-MCBS score is downgraded if toxicity is increased or QoL analysis does not show an improvement. Obviously, if mature OS data or QoL results are not published, as is yet still the case for most PARP inhibitor trials, this downgrade will not be applied. ESMO-MCBS forms do not currently allow downgrading when OS and QoL data are not published, but this could be relevant in cases in which sufficient time has passed for data to become mature and publication of OS data is no longer expected to follow. In ovarian cancer trials, a PFS benefit often does not predict a benefit in OS.^43^ However, mature OS data can be difficult to interpret because of crossover. Furthermore, a difference in sensitivity to reintroduction of some chemotherapies in responders versus non-responders to PARP inhibitors due to a difference in DNA damage repair capability further complicates ascribing an observed difference in OS to a single treatment regimen. An improvement of PFS accompanied by an improvement in QoL could be of clinical value for patients, even in the case that no OS benefit is established.

Downgrading for toxicity can be a matter of debate. For example, in the AURELIA trial, global QoL was comparable but there was an improvement in a subscale of QoL measuring GI symptoms.^31^ This subscale analysis was predefined and therefore the preliminary score of 3 was upgraded to a 4. The additional toxicity of bevacizumab, such as an extra 2% GI perforation (grade 2 or higher), arterial thromboembolic events and fistula/abscess formation should be taken into account in the benefit/risk evaluation of this treatment, although this does not meet the criteria for a downgrade of the ESMO-MCBS score.

There are some other points to consider in interpreting the current analysis. First of all, studies from 1990 onwards were included. Surgical treatment and supportive care have changed during this time period, complicating the comparison of study results within these three decades. Furthermore, the ESMO-MCBS was developed to grade the results of well-designed registration studies, and the standards for performing and reporting of clinical trials have become more stringent during the years. However, even in recent times, only a minority of studies testing anticancer drugs were adequately designed to be able to meet ESMO-MCBS thresholds of substantial clinical benefit.^44^ When head-to-head comparisons testing superiority for different treatments are not available, and the studies testing those treatments used different comparator arms, it is complicated to compare ESMO-MCBS scores of these treatments. An example is the use of carboplatin/paclitaxel (grade 4, compared to carboplatin) versus carboplatin/PLD (grade 3, compared to carboplatin/paclitaxel in a non-inferiority study) in the platinum-sensitive setting. Despite the lower ESMO-MCBS grade, one could argue that carboplatin/PLD is the better combination given the stronger comparator arm in this study. Therefore, when taking into account ESMO-MCBS grading for treatment decision making, it is crucial to critically assess differences in comparators used between studies. We attempted to give insight into the relative benefit of various head-to-head comparisons tested in the platinum-sensitive (Figure 2A) and platinum-resistant (Figure 2B) setting, but this should be interpreted with caution given the heterogeneity between studies. Finally, more detailed consideration of toxicity, impact on QoL and the optimal sequences of therapies on an individual patient level should be taken into account to make treatment decisions.

### Conclusion

Only a few licensed systemic therapies for recurrent ovarian cancer show substantial clinical benefit based on ESMO-MCBS scores. Carboplatin + paclitaxel scores a grade 4 indicating substantial clinical benefit in the platinum-sensitive setting. Addition of bevacizumab to either PLD, paclitaxel or topotecan in platinum-resistant disease also has substantial benefit (grade 4), but the QoL data supporting this score are subject to interpretation. The unlicensed WEE1 inhibitor adavosertib combined with gemcitabine has a preliminary score of 4 based on an OS benefit observed in a phase II study. For PARP inhibitors, only moderate clinical benefit can be confirmed, based on OS results for olaparib tablets and PFS results for the other drugs.

## References

[1] Torre L.A., Trabert B., DeSantis C.E. (2018). Ovarian cancer statistics, 2018. CA Cancer J Clin.

[2] Colombo N., Sessa C., du Bois A. (2019). ESMO-ESGO consensus conference recommendations on ovarian cancer: pathology and molecular biology, early and advanced stages, borderline tumours and recurrent disease. Ann Oncol.

[3] ESMO Guidelines Committee eUpdate 2020—Relapsed epithelial ovarian carcinoma treatment recommendations. https://www.esmo.org/guidelines/gynaecological-cancers/newly-diagnosed-and-relapsed-epithelial-ovarian-carcinoma/eupdate-ovarian-cancer-treatment-recommendations.

[4] Cherny N.I., Dafni U., Bogaerts J. (2017). ESMO-Magnitude of Clinical Benefit Scale version 1.1. Ann Oncol.

[5] Pignata S., Scambia G., Bologna A. (2017). Randomized controlled trial testing the efficacy of platinum-free interval prolongation in advanced ovarian cancer: the MITO-8, MaNGO, BGOG-Ov1, AGO-Ovar2.16, ENGOT-Ov1, GCIG study. J Clin Oncol.

[6] ESMO-Magnitude of Clinical Benefit Scale: scale evaluation forms V1.0 and V1.1. https://www.esmo.org/guidelines/esmo-mcbs/scale-evaluation-forms-v1.0-v1.1.

[7] Parmar M.K.B., Ledermann J.A., Colombo N. (2003). Paclitaxel plus platinum-based chemotherapy versus conventional platinum-based chemotherapy in women with relapsed ovarian cancer: the ICON4/AGO-OVAR-2.2 trial. Lancet.

[8] Pujade Lauraine E., Wagner U., Aavall Lundqvist E. (2010). Pegylated liposomal doxorubicin and carboplatin compared with paclitaxel and carboplatin for patients with platinum-sensitive ovarian cancer in late relapse. J Clin Oncol.

[9] Brundage M., Gropp M., Mefti F. (2012). Health-related quality of life in recurrent platinum-sensitive ovarian cancer-results from the CALYPSO trial. Ann Oncol.

[10] Pfisterer J., Plante M., Vergote I. (2006). Gemcitabine plus carboplatin compared with carboplatin in patients with platinum-sensitive recurrent ovarian cancer: an intergroup trial of the AGO-OVAR, the NCIC CTG, and the EORTC GCG. J Clin Oncol.

[11] Pfisterer J., Shannon C.M., Baumann K. (2020). Bevacizumab and platinum-based combinations for recurrent ovarian cancer: a randomised, open-label, phase 3 trial. Lancet Oncol.

[12] Pignata S., Lorusso D., Joly F. (2021). Carboplatin-based doublet plus bevacizumab beyond progression versus carboplatin-based doublet alone in patients with platinum-sensitive ovarian cancer: a randomised, phase 3 trial. Lancet Oncol.

[13] Vergote I., Armstrong D., Scambia G. (2016). A randomized, double-blind, placebo-controlled, phase III study to assess efficacy and safety of weekly farletuzumab in combination with carboplatin and taxane in patients with ovarian cancer in first platinum-sensitive relapse. J Clin Oncol.

[14] Sehouli J., Chekerov R., Reinthaller A. (2016). Topotecan plus carboplatin versus standard therapy with paclitaxel plus carboplatin (PC) or gemcitabine plus carboplatin (GC) or pegylated liposomal doxorubicin plus carboplatin (PLDC): a randomized phase III trial of the NOGGO-AGO-Study Group-AGO Austria and GEICO-ENGOT-GCIG intergroup study (HECTOR). Ann Oncol.

[15] Vergote I., Bergfeldt K., Franquet A. (2020). A randomized phase III trial in patients with recurrent platinum sensitive ovarian cancer comparing efficacy and safety of paclitaxel micellar and Cremophor EL-paclitaxel. Gynecol Oncol.

[16] Wagner U., Marth C., Largillier R. (2012). Final overall survival results of phase III GCIG CALYPSO trial of pegylated liposomal doxorubicin and carboplatin vs paclitaxel and carboplatin in platinum-sensitive ovarian cancer patients. Br J Cancer.

[17] Mirza M.R., Monk B.J., Herrstedt J. (2016). Niraparib maintenance therapy in platinum-sensitive, recurrent ovarian cancer. N Engl J Med.

[18] Coleman R.L., Oza A.M., Lorusso D. (2017). Rucaparib maintenance treatment for recurrent ovarian carcinoma after response to platinum therapy (ARIEL3): a randomised, double-blind, placebo-controlled phase 3 trial. Lancet.

[19] Ledermann J.A., Harter P., Gourley C. (2016). Overall survival in patients with platinum-sensitive recurrent serous ovarian cancer receiving olaparib maintenance monotherapy: an updated analysis from a randomised, placebo-controlled, double-blind, phase 2 trial. Lancet Oncol.

[20] Pujade-Lauraine E., Ledermann J.A., Selle F. (2017). Olaparib tablets as maintenance therapy in patients with platinum-sensitive, relapsed ovarian cancer and a BRCA1/2 mutation (SOLO2/ENGOT-Ov21): a double-blind, randomised, placebo-controlled, phase 3 trial. Lancet Oncol.

[21] Ledermann J.A., Embleton A.C., Raja F. (2016). Cediranib in patients with relapsed platinum-sensitive ovarian cancer (ICON6): a randomised, double-blind, placebo-controlled phase 3 trial. Lancet.

[22] Aghajanian C., Blank S.V., Goff B.A. (2012). OCEANS: a randomized, double-blind, placebo-controlled phase III trial of chemotherapy with or without bevacizumab in patients with platinum-sensitive recurrent epithelial ovarian, primary peritoneal, or fallopian tube cancer. J Clin Oncol.

[23] Aghajanian C., Goff B.A., Nycum L.R. (2015). Final overall survival and safety analysis of OCEANS, a phase 3 trial of chemotherapy with or without bevacizumab in patients with platinum-sensitive recurrent ovarian cancer. Gynecol Oncol.

[24] Friedlander M., Matulonis U., Gourley C. (2018). Long-term efficacy, tolerability and overall survival in patients with platinum-sensitive, recurrent high-grade serous ovarian cancer treated with maintenance olaparib capsules following response to chemotherapy. Br J Cancer.

[25] Oza A.M., Cibula D., Benzaquen A.O. (2015). Olaparib combined with chemotherapy for recurrent platinum-sensitive ovarian cancer: a randomised phase 2 trial. Lancet Oncol.

[26] Coleman R.L., Brady M.F., Herzog T.J. (2017). Bevacizumab and paclitaxel-carboplatin chemotherapy and secondary cytoreduction in recurrent, platinum-sensitive ovarian cancer (NRG Oncology/Gynecologic Oncology Group study GOG-0213): a multicentre, open-label, randomised, phase 3 trial. Lancet Oncol.

[27] Poveda A., Floquet A., Ledermann J.A. (2021). Olaparib tablets as maintenance therapy in patients with platinum-sensitive relapsed ovarian cancer and a BRCA1/2 mutation (SOLO2/ENGOT-Ov21): a final analysis of a double-blind, randomised, placebo-controlled, phase 3 trial. Lancet Oncol.

[28] Ledermann J.A., Embleton-Thirsk A.C., Perren T.J. (2021). Cediranib in addition to chemotherapy for women with relapsed platinum-sensitive ovarian cancer (ICON6): overall survival results of a phase III randomised trial. ESMO Open.

[29] Kaye S.B., Fehrenbacher L., Holloway R. (2012). A phase II, randomized, placebo-controlled study of vismodegib as maintenance therapy in patients with ovarian cancer in second or third complete remission. Clin Cancer Res.

[30] Pujade Lauraine E., Hilpert F., Weber B. (2014). Bevacizumab combined with chemotherapy for platinum-resistant recurrent ovarian cancer: the AURELIA open-label randomized phase III trial. J Clin Oncol.

[31] Stockler M.R., Hilpert F., Friedlander M. (2014). Patient-reported outcome results from the open-label phase III AURELIA trial evaluating bevacizumab-containing therapy for platinum-resistant ovarian cancer. J Clin Oncol.

[32] Lheureux S., Cristea M.C., Bruce J.P. (2021). Adavosertib plus gemcitabine for platinum-resistant or platinum-refractory recurrent ovarian cancer: a double-blind, randomised, placebo-controlled, phase 2 trial. Lancet.

[33] Chekerov R., Hilpert F., Mahner S. (2018). Sorafenib plus topotecan versus placebo plus topotecan for platinum-resistant ovarian cancer (TRIAS): a multicentre, randomised, double-blind, placebo-controlled, phase 2 trial. Lancet Oncol.

[34] Monk B., Herzog T., Kaye S. (2010). Trabectedin plus pegylated liposomal doxorubicin in recurrent ovarian cancer. J Clin Oncol.

[35] Monk B., Herzog T., Kaye S. (2012). Trabectedin plus pegylated liposomal doxorubicin (PLD) versus PLD in recurrent ovarian cancer: overall survival analysis. Eur J Cancer.

[36] Monk B., Poveda A., Vergote I. (2014). Anti-angiopoietin therapy with trebananib for recurrent ovarian cancer (TRINOVA-1): a randomised, multicentre, double-blind, placebo-controlled phase 3 trial. Lancet Oncol.

[37] Konstantinopoulos P.A., Cheng S., Wahner Hendrickson A.E. (2020). Berzosertib plus gemcitabine versus gemcitabine alone in platinum-resistant high-grade serous ovarian cancer: a multicentre, open-label, randomised, phase 2 trial. Lancet Oncol.

[38] Wu J., Sun H., Yang L. (2018). Improved survival in ovarian cancer, with widening survival gaps of races and socioeconomic status: a period analysis, 1983-2012. J Cancer.

[39] Helleday T. (2011). The underlying mechanism for the PARP and BRCA synthetic lethality: clearing up the misunderstandings. Mol Oncol.

[40] Takaya H., Nakai H., Takamatsu S. (2020). Homologous recombination deficiency status-based classification of high-grade serous ovarian carcinoma. Sci Rep.

[41] Berchuck A., Secord A.A., Moss H.A., Havrilesky L.J. (2017). Maintenance poly (ADP-ribose) polymerase inhibitor therapy for ovarian cancer: precision oncology or one size fits all?. J Clin Oncol.

[42] European Public Assessment Report for Lynparza tablets. https://www.ema.europa.eu/en/documents/variation-report/lynparza-h-c-3726-x-0016-g-epar-assessment-report-extension_en.pdf.

[43] Paoletti X., Lewsley L.A., Daniele G. (2020). Assessment of progression-free survival as a surrogate end point of overall survival in first-line treatment of ovarian cancer: a systematic review and meta-analysis. JAMA Netw Open.

[44] Del Paggio J.C., Azariah B., Sullivan R. (2017). Do contemporary randomized controlled trials meet ESMO thresholds for meaningful clinical benefit?. Ann Oncol.

[45] ten Bokkel Huinink W., Gore M., Carmichael J. (1997). Topotecan versus paclitaxel for the treatment of recurrent epithelial ovarian cancer. J Clin Oncol.

[46] Gordon A.N., Fleagle J.T., Guthrie D. (2001). Recurrent epithelial ovarian carcinoma: a randomized phase III study of pegylated liposomal doxorubicin versus topotecan. J Clin Oncol.

[47] Gordon A.N., Tonda N., Sun S. (2004). Long-term survival advantage for women treated with pegylated liposomal doxorubicin compared with topotecan in a phase 3 randomized study of recurrent and refractory epithelial ovarian cancer. Gynecol Oncol.

[48] Buda A., Floriani I., Rossi R. (2004). Randomised controlled trial comparing single agent paclitaxel vs epidoxorubicin plus paclitaxel in patients with advanced ovarian cancer in early progression after platinum-based chemotherapy: an Italian Collaborative Study from the Mario Negri Institute, Milan, G.O.N.O. (Gruppo Oncologico Nord Ovest) group and I.O.R. (Istituto Oncologico Romagnolo) group. Br J Cancer.

[49] Mutch D., Orlando M., Goss T. (2007). Randomized phase III trial of gemcitabine compared with pegylated liposomal doxorubicin in patients with platinum-resistant ovarian cancer. J Clin Oncol.

[50] Ferrandina G., Ludovisi M., Lorusso D. (2008). Phase III trial of gemcitabine compared with pegylated liposomal doxorubicin in progressive or recurrent ovarian cancer. J Clin Oncol.

[51] Sehouli J., Stengel D., Oskay Oezcelik G. (2008). Nonplatinum topotecan combinations versus topotecan alone for recurrent ovarian cancer: results of a phase III study of the North-Eastern German Society of Gynecological Oncology Ovarian Cancer Study Group. J Clin Oncol.

[52] Vergote I., Finkler N., del Campo J. (2009). Phase 3 randomised study of canfosfamide (Telcyta, TLK286) versus pegylated liposomal doxorubicin or topotecan as third-line therapy in patients with platinum-refractory or -resistant ovarian cancer. Eur J Cancer.

[53] Makhija S., Amler L.C., Glenn D. (2010). Clinical activity of gemcitabine plus pertuzumab in platinum-resistant ovarian cancer, fallopian tube cancer, or primary peritoneal cancer. J Clin Oncol.

[54] Kaye S.B., Poole C.J., Danska-Bidzinska A. (2013). A randomized phase II study evaluating the combination of carboplatin-based chemotherapy with pertuzumab versus carboplatin-based therapy alone in patients with relapsed, platinum-sensitive ovarian cancer. Ann Oncol.

[55] Lortholary A., Largillier R., Weber B. (2012). Weekly paclitaxel as a single agent or in combination with carboplatin or weekly topotecan in patients with resistant ovarian cancer: the CARTAXHY randomized phase II trial from Groupe d'Investigateurs Nationaux pour l'Etude des Cancers Ovariens (GINECO). Ann Oncol.

[56] Kaye S.B., Lubinski J., Matulonis U. (2012). Phase II, open-label, randomized, multicenter study comparing the efficacy and safety of olaparib, a poly (ADP-ribose) polymerase inhibitor, and pegylated liposomal doxorubicin in patients with BRCA1 or BRCA2 mutations and recurrent ovarian cancer. J Clin Oncol.

[57] Colombo N., Kutarska E., Dimopoulos M. (2012). Randomized, open-label, phase III study comparing patupilone (EPO906) with pegylated liposomal doxorubicin in platinum-refractory or -resistant patients with recurrent epithelial ovarian, primary fallopian tube, or primary peritoneal cancer. J Clin Oncol.

[58] Coleman R.L., Moon J., Sood A.K. (2014). Randomised phase II study of docetaxel plus vandetanib versus docetaxel followed by vandetanib in patients with persistent or recurrent epithelial ovarian, fallopian tube or primary peritoneal carcinoma: SWOG S0904. Eur J Cancer.

[59] Liu J.F., Ray-Coquard I., Selle F. (2016). Randomized phase II trial of seribantumab in combination with paclitaxel in patients with advanced platinum-resistant or -refractory ovarian cancer. J Clin Oncol.

[60] Monk B.J., Brady M.F., Aghajanian C. (2017). A phase 2, randomized, double-blind, placebo- controlled study of chemo-immunotherapy combination using motolimod with pegylated liposomal doxorubicin in recurrent or persistent ovarian cancer: a Gynecologic Oncology Group partners study. Ann Oncol.

[61] Cohn D.E., Sill M.W., Walker J.L. (2017). Randomized phase IIB evaluation of weekly paclitaxel versus weekly paclitaxel with oncolytic reovirus (Reolysin®) in recurrent ovarian, tubal, or peritoneal cancer: an NRG Oncology/Gynecologic Oncology Group study. Gynecol Oncol.

[62] Richardson D.L., Sill M.W., Coleman R.L. (2018). Paclitaxel with and without pazopanib for persistent or recurrent ovarian cancer: a randomized clinical trial. JAMA Oncol.

[63] Matulonis U.A., Sill M.W., Makker V. (2019). A randomized phase II study of cabozantinib versus weekly paclitaxel in the treatment of persistent or recurrent epithelial ovarian, fallopian tube or primary peritoneal cancer: an NRG Oncology/Gynecologic Oncology Group study. Gynecol Oncol.

[64] McGuire W.P., Penson R.T., Gore M. (2018). Randomized phase II study of the PDGFRα antibody olaratumab plus liposomal doxorubicin versus liposomal doxorubicin alone in patients with platinum-refractory or platinum-resistant advanced ovarian cancer. BMC Cancer.

[65] Oza A.M., Kaye S., Van Tornout J. (2018). Phase 2 study evaluating intermittent and continuous linsitinib and weekly paclitaxel in patients with recurrent platinum resistant ovarian epithelial cancer. Gynecol Oncol.

[66] Falchook G., Coleman R.L., Roszak A. (2019). Alisertib in combination with weekly paclitaxel in patients with advanced breast cancer or recurrent ovarian cancer: a randomized clinical trial. JAMA Oncol.

[67] Oza A.M., Matulonis U.A., Alvarez Secord A. (2020). A randomized phase II trial of epigenetic priming with guadecitabine and carboplatin in platinum-resistant, recurrent ovarian cancer. Clin Cancer Res.

