# Supplementary material for: Clinical benefit of systemic therapies for recurrent ovarian cancer—ESMO-MCBS scores
Source: ESMO Open. 2021 Aug 7;6(4):100229. doi: 10.1016/j.esmoop.2021.100229 (PMC8358417; doi:10.1016/j.esmoop.2021.100229)
Supplement: Supplementary Table S1 [file mmc1.docx]

**Supplementary Table 1: RCTs excluded from the analysis, *n*=29**

|  | **Treatment** | **Study name** | | ***N*** | **Control** | **Design** | **Primary end point** | **PFS control** | | **PFS gain** | **HR** | **OS control** | **OS gain** | **OS HR** | **ORR** | **Toxicity/QoL** | **Reason for exclusion** | **Ref** |  |
| --- | --- | --- | --- | --- | --- | --- | --- | --- | --- | --- | --- | --- | --- | --- | --- | --- | --- | --- | --- |
|  | **Platinum-sensitive recurrence** | | | | | | | | | | | | | | | | | |  |
|  | Cyclophosphamide, doxorubicin, cisplatin (CAP) | |  | 97 | Paclitaxel | RCT | OS |  | |  |  | 25.8 | 8.9 | 0.70  (0.42-1.15) |  |  | Suboptimal (non-platinum) control arm | [1] |  |
|  | Carboplatin plus PLD | |  | 61 | Carboplatin | Phase II RCT | OS |  | |  |  | 18.0 | 8.0 | 0.46  (0.22-0.95) |  |  | Terminated prematurely  Phase III CALYPSO study graded | [2] |  |
|  | Carboplatin plus PLD | |  | 189 | Carboplatin plus paclitaxel | Phase II RCT | ORR |  | |  |  |  |  |  | ORR 57% vs 51%, NS |  | Phase III CALYPSO study graded | [3] |  |
|  | Docetaxel plus carboplatin | |  | 150 | Sequential docetaxel - carboplatin | RCT | PFS to historical controls |  | |  |  |  |  |  |  |  | Due to slow accrual endpoint amended to compare PFS for each arm separately to historic controls | [4] |  |
|  | PLD plus trabectedin | |  | 576 | PLD | Phase III RCT | OS |  | |  |  | 22.2 | 1.6 | 0.92  (0.73-1.18) |  |  | Suboptimal (non-platinum) control arm | [5] |  |
|  | Niraparib plus bevacizumab | |  | 97 | Niraparib | Phase II RCT | PFS | 5.5 | | 6.4 | 0.35  (0.21-0.57) |  |  |  |  |  | Suboptimal (non-platinum) control arm | [6] |  |
|  | **Maintenance therapy after response to 2nd line platinum-based chemotherapy** | | | | | | | | | | | | | | | | | |  |
|  | Niraparib | |  | 265 | placebo | Phase III  RCT | PFS | 5.4 | | 12.9 | 0.32  (0.23-0.45) |  |  |  |  |  | Larger phase III study available | [7] |  |
|  | **Platinum-resistant recurrence** | | | | | | | | | | | | | | | | | |  |
|  | Topotecan # | |  | 274 | Treosulfan | Phase III RCT | OS |  | |  |  | 9.5 | 3.2 | 0.67  (0.52-0.87) |  |  | Suboptimal control arm | [8] |  |
|  | Paclitaxel 250 mg/m^2^ # | |  | 330 | Paclitaxel 175 mg/m^2^ | Phase III RCT | OS |  | |  |  | 12.3 | 0.8 | 0.97  (0.77-1.22) |  |  | Suboptimal control arm | [9] |  |
|  | Etirinotecan pegol every 14 days | |  | 71 | Etirinotecan pegol every 21 days | Phase II RCT | ORR |  | |  |  |  |  |  | ORR 20% for Q2W regimen | -1 for incremental toxicity (70% gr 3-4 AE’s) | ESMO MCBS 1-1 =0  Study lacks active control | [10] |  |
|  | Treosulfan | |  | 78 | Leuprorelin | Phase III RCT | OS |  | |  |  | 6.9 | 1.4 | 0.98  (0.58-1.67) |  |  | Terminated prematurely | [11] |  |
|  | Treosulfan orally | |  | 250 | Treosulfan intravenously | Phase III RCT | Safety |  | |  |  |  |  |  |  |  | Primary end point not meeting criteria for grading | [12] |  |
|  | Pemetrexed 500mg/m2 | |  | 102 | Pemetrexed 900 mg/m2 | Phase II  RCT | Safety |  | |  |  |  |  |  |  |  | Primary end point not meeting criteria for grading | [13] |  |
|  | Trebananib 3 mg/kg or 10 mg/kg plus paclitaxel |  | | 161 | Placebo | Phase II RCT | PFS | | 4.6 | 1.1-2.6 | 0.76  (0.52-1.12 |  |  |  |  |  | Study lacks active control | [14] |  |
|  | Vintafolide plus PLD | PRECEDENT | | 149 | PLD | Phase II RCT | PFS | | 2.7 | 2.3 | 0.63  (0.41-0.96) |  |  |  |  |  | Confirmatory phase III terminated | [15] |  |
|  | Secondary CRS plus HIPEC |  | | 120 | Secondary CRS without HIPEC | Phase III RCT | OS | |  |  |  | 13.4 | 13.3 | Not provided |  | No toxicity data provided | HR not provided and lacks toxicity data | [16] |  |
|  | Weekly carboplatin plus phenoxodiol | OVATURE | | 142 | Weekly carboplatin plus placebo | Phase III  RCT | PFS | | 3.6 | 1.0 | 1.22  (0.84-1.22) |  |  |  |  |  | Terminated prematurely | [17] |  |
|  | Leuprorelin |  | | 78 | Treosulfan | RCT | OS | |  |  |  | 6.9 | 1.4 | 0.98  (0.58-1.67) |  |  | Terminated prematurely | [11] |  |
|  | Paclitaxel plus epidoxorubicin |  | | 81 | Paclitaxel | RCT | ORR | |  |  |  |  |  |  | ORR 34% vs. 17% |  | Phase III trial available | [18] |  |
|  | Topotecan weekly |  | | 194 | Topotecan conventional 5-day dose regimen | RCT | ORR | |  |  |  |  |  |  | ORR 0% |  | Dose-finding study | [19] |  |
|  | Aflibercept 2 mg/kg |  | | 215 | Aflibercept 4 mg/mkg | RCT | ORR | |  |  |  |  |  |  | ORR 0.9% vs. 4.6% |  | Dose-finding study | [20] |  |
|  | Topotecan plus thalidomide |  | | 75 | Topotecan | Phase II RCT | ORR | |  |  |  |  |  |  | ORR 47% vs. 21% |  | Terminated prematurely, included also CA-125 response | [21] |  |
|  | Canfosfamide plus PLD |  | | 125 | PLD | Phase III RCT | PFS | | 3.7 | 1.9 | 0.92, NS |  |  |  |  |  | Terminated prematurely | [22] |  |
|  | Everolimus plus bevacizumab |  | | 150 | Bevacizumab | RCT | PFS | | 4.5 | 1.4 | 0.95  (0.66-1.37) |  |  |  |  |  | Lacks standard control arm | [23] |  |
|  | Thalidomide |  | | 139 | Tamoxifen | RCT | PFS | | 4.5 | -1.3 | 1.31  (0.93-1.85) | 33.2 | -9.2 | 1.76  (1.16-2.68) |  |  | Terminated prematurely due to futility | [24] |  |
|  | Volasertib |  | | 109 | Investigator choice non-platinum chemotherapy | Phase II  RCT | DoR | |  |  |  |  |  |  |  |  | Primary endpoint not meeting criteria for grading | [25] |  |
|  | Gemcitabine plus pazopanib# |  | | 148 | Gemcitabine | Phase II RCT | PFS | | 2.9 | 2.4 | 0.61  (0.40-0.92) |  |  |  |  |  | Large subgroup platinum-sensitive (40%), lacks standard control arm | [26] |  |
|  | Nivolumab plus ipilimumab# |  | | 100 | Nivolumab | Phase II RCT | PFS | | 2.0 | 1.9 | 0.53  (0.34-0.82) |  |  |  |  |  | Lacks standard control arm | [27] |  |
|  | Belotecan# |  | | 141 | Topotecan | Phase II  RCT | ORR | |  |  |  |  |  |  | ORR 29.6 vs 26.1 % |  | Inadequate comparator | [28] |  |
|  | # trial did also include platinum-sensitive patients | | | | | | | | | | | | | | | | | |  |

References

1. Cantu MG, Buda A, Parma G, et al. Randomized controlled trial of single-agent paclitaxel versus cyclophosphamide, doxorubicin, and cisplatin in patients with recurrent ovarian cancer who responded to first-line platinum-based regimens. *J Clin Oncol.* 2002;20:1232-1237.
2. Alberts DS, Liu PY, Wilczinsky SP, et al. Randomized trial of pegylated liposomal doxorubicin (PLD) plus carboplatin versus carboplatin in platinum-sensitive (PS) patients with recurrent epithelial ovarian or peritoneal carcinoma after failure of initial platinum-based chemotherapy (Southwest Oncology Group Protocol S0200). *Gynecol Oncol.* 2008;108:90-94.
3. Bafaloukos D, Linardou H, Aravantinos G, et al. A randomized phase II study of carboplatin plus pegylated liposomal doxorubicin versus carboplatin plus paclitaxel in platinum sensitive ovarian cancer patients: a Hellenic Cooperative Oncology Group study. *BMC Med.* 2010;8:3-3.
4. Alvarez Secord A, Berchuck A, Higgins RV, et al. A multicenter, randomized, phase 2 clinical trial to evaluate the efficacy and safety of combination docetaxel and carboplatin and sequential therapy with docetaxel then carboplatin in patients with recurrent platinum-sensitive ovarian cancer. *Cancer.* 2012;118:3283-3293.
5. Monk BJ, Herzog TJ, Wang G, et al. A phase 3 randomized, open-label, multicenter trial for safety and efficacy of combined trabectedin and pegylated liposomal doxorubicin therapy for recurrent ovarian cancer. *Gynecol Oncol.* 2020;156:535-544.
6. Mirza MR, Åvall Lundqvist E, Birrer MJ, et al. Niraparib plus bevacizumab versus niraparib alone for platinum-sensitive recurrent ovarian cancer (NSGO-AVANOVA2/ENGOT-ov24): a randomised, phase 2, superiority trial. *Lancet Oncol.* 2019;20:1409-1419.
7. Wu XH, Zhu JQ, Yin RT, et al. Niraparib maintenance therapy in patients with platinum-sensitive recurrent ovarian cancer using an individualized starting dose (NORA): a randomized, double-blind, placebo-controlled phase III trial. *Ann Oncol.* 2021;32:512-521.
8. Meier W, du Bois A, Reuss A, et al. Topotecan versus treosulfan, an alkylating agent, in patients with epithelial ovarian cancer and relapse within 12 months following 1st-line platinum/paclitaxel chemotherapy. A prospectively randomized phase III trial by the Arbeitsgemeinschaft Gynaekologische Onkologie Ovarian Cancer Study Group (AGO-OVAR). *Gynecol Oncol.* 2009;114:199-205.
9. Omura GA, Brady MF, Look KY, et al. Phase III trial of paclitaxel at two dose levels, the higher dose accompanied by filgrastim at two dose levels in platinum-pretreated epithelial ovarian cancer: an intergroup study. *J Clin Oncol.* 2003;21:2843-2848.
10. Vergote IB, Garcia A, Micha J, et al. Randomized multicenter phase II trial comparing two schedules of etirinotecan pegol (NKTR-102) in women with recurrent platinum-resistant/refractory epithelial ovarian cancer. *J Clin Oncol.* 2013;31:4060-4066.
11. du Bois A, Meier W, Luck HJ, et al. Chemotherapy versus hormonal treatment in platinum- and paclitaxel-refractory ovarian cancer: a randomised trial of the German Arbeitsgemeinschaft Gynaekologische Onkologie (AGO) Study Group Ovarian Cancer. *Ann Oncol.* 2002;13:251-257.
12. Sehouli J, Tome O, Dimitrova D, et al. A phase III, open label, randomized multicenter controlled trial of oral versus intravenous treosulfan in heavily pretreated recurrent ovarian cancer: a study of the North-Eastern German Society of Gynecological Oncology (NOGGO). *J Cancer Res Clin Oncol.* 2017;143:541-550.
13. Vergote I, Calvert H, Kania M, et al. A randomised, double-blind, phase II study of two doses of pemetrexed in the treatment of platinum-resistant, epithelial ovarian or primary peritoneal cancer. *Eur J Cancer.* 2009; 45:1415-1423.
14. Karlan BY, Oza AM, Richardson GE, et al. Randomized, double-blind, placebo-controlled phase II study of AMG 386 combined with weekly paclitaxel in patients with recurrent ovarian cancer. *J Clin Oncol.* 2012;30:362-371.
15. Naumann RW, Coleman RL, Burger RA, et al. PRECEDENT: a randomized phase II trial comparing vintafolide (EC145) and pegylated liposomal doxorubicin (PLD) in combination versus PLD alone in patients with platinum-resistant ovarian cancer. *J Clin Oncol.* 2013;31:4400-4406.
16. Spiliotis J, Halkia E, Lianos E, et al. Cytoreductive surgery and HIPEC in recurrent epithelial ovarian cancer: a prospective randomized phase III study. *Ann Surg Oncol.* 2015;22:1570-1575.
17. Fotopoulou C, Vergote I, Mainwaring P, et al. Weekly AUC2 carboplatin in acquired platinum-resistant ovarian cancer with or without oral phenoxodiol, a sensitizer of platinum cytotoxicity: the phase III OVATURE multicenter randomized study. *Ann Oncol.* 2014;25:160-165.
18. Bolis G, Parazzini F, Scarfone G, et al. Paclitaxel vs epidoxorubicin plus paclitaxel as second-line therapy for platinum-refractory and -resistant ovarian cancer. *Gynecol Oncol.* 1999;72:60-64.
19. Sehouli J, Stengel D, Harter P, et al. Topotecan weekly versus conventional 5-day schedule in patients with platinum-resistant ovarian cancer: a randomized multicenter phase II trial of the North-Eastern German Society of Gynecological Oncology Ovarian Cancer Study Group. *J Clin Oncol.* 2011;29:242-248.
20. Tew WP, Colombo N, Ray-Coquard I, et al. Intravenous aflibercept in patients with platinum-resistant, advanced ovarian cancer: results of a randomized, double-blind, phase 2, parallel-arm study. *Cancer.* 2014;120:335-343.
21. Downs LS Jr, Judson PL, Argenta PA, et al. A prospective randomized trial of thalidomide with topotecan compared with topotecan alone in women with recurrent epithelial ovarian carcinoma. *Cancer.* 2008;112:331-339.
22. Vergote I, Finkler N, Hall J, et al. Randomized phase III study of canfosfamide in combination with pegylated liposomal doxorubicin compared with pegylated liposomal doxorubicin alone in platinum-resistant ovarian cancer. *Int J Gynecol Cancer.* 2010;20:772-780.
23. Tew WP, Sill MW, Walker JL, et al. Randomized phase II trial of bevacizumab plus everolimus versus bevacizumab alone for recurrent or persistent ovarian, fallopian tube or peritoneal carcinoma: an NRG oncology/gynecologic oncology group study. *Gynecol Oncol.* 2018;151:257-263.
24. Hurteau J, Brady M, Darcy K, et al. Randomized phase III trial of tamoxifen versus thalidomide in women with biochemical-recurrent-only epithelial ovarian, fallopian tube or primary peritoneal carcinoma after a complete response to first-line platinum/taxane chemotherapy with an evaluation of serum vascular endothelial growth factor (VEGF): A Gynecologic Oncology Group Study. *Gynecol Oncol.* 2010;119:444-450.
25. Pujade-Lauraine E, Selle F, Weber B, et al. Volasertib versus chemotherapy in platinum-resistant or -refractory ovarian cancer: a randomized phase II groupe des investigateurs nationaux pour l'etude des cancers de l'ovaire study. *J Clin Oncol.* 2016;34:706-713.
26. Duska LR, Petroni GR, Varhegyi N, et al. A randomized phase II evaluation of weekly gemcitabine plus pazopanib versus weekly gemcitabine alone in the treatment of persistent or recurrent epithelial ovarian, fallopian tube or primary peritoneal carcinoma. *Gynecol Oncol.* 2020;157:585-592.
27. Zamarin D, Burger RA, Sill MW, et al. Randomized phase II trial of nivolumab versus nivolumab and ipilimumab for recurrent or persistent ovarian cancer: an NRG oncology study. *J Clin Oncol.* 2020;38:1814-1823.
28. Kim HS, Park SY, Park CY, et al. A multicentre, randomised, open-label, parallel-group Phase 2b study of belotecan versus topotecan for recurrent ovarian cancer. *Br J Cancer.* 2021;124:375-382.
